# Postmortem Chest Computed Tomography in Fatal COVID-19: A Valuable Diagnostic Tool for Minimally Invasive Autopsy

**DOI:** 10.6061/clinics/2021/e3551

**Published:** 2021-11-24

**Authors:** Paulo Savoia Dias da Silva, Marcio Valente Yamada Sawamura, Renata Aparecida de Almeida Monteiro, Amaro Nunes Duarte-Neto, Maria da Graça Morais Martin, Marisa Dolhnikoff, Thais Mauad, Paulo Hilário Nascimento Saldiva, Claudia Costa Leite, Luiz Fernando Ferraz da Silva, Ellison Fernando Cardoso

**Affiliations:** I Faculdade de Medicina FMUSP, Universidade de Sao Paulo, Sao Paulo, SP, BR.; II Hospital Israelita Albert Einstein, Sao Paulo, SP, BR.; III Fleury Group, Sao Paulo, SP, BR.

The coronavirus disease (COVID-19) pandemic has resulted in more than 4.7 million deaths worldwide (1). Despite the high number of COVID-19-related deaths, published reports on autopsies are scarce, probably because of contagion risk and/or recommended strict protection procedures that restrict autopsies considerably (2,3). Therefore, to address this postmortem knowledge gap, some authors have studied patients who died because of COVID-19 using minimally invasive autopsy methods but not chest computed tomography (CT) (4,5).

Postmortem chest CT has some limitations owing to the inherent characteristics at death such as expired lungs and hypostasis. However, we believe these characteristics do not significantly limit the value of postmortem CT, especially during the COVID-19 pandemic, when traditional autopsies are often avoided or even forbidden.

Few authors have used postmortem chest CT to study fatal COVID-19, but some case reports have been published (6-9). Helmrich et al. presented a case series in which postmortem chest CT was used as a triage tool to refer a body for conventional autopsy when no typical CT characteristics of COVID-19 were found (10). De-Giorgio et al. used postmortem chest CT to confirm or exclude the disease and minimize risks of contagion to the autopsy team (11). Both studies suggest that postmortem CT is especially useful when reverse transcription polymerase chain reaction (RT-PCR) is not feasible.

To validate postmortem chest CT findings, we selected the 5 of 117 patients who had a premortem chest CT performed at most 2 days before death to compare their premortem with their postmortem chest CT and describe findings as well as eventual associated conditions. We hypothesized that a postmortem chest CT could help us understand and stage COVID-19, as well as diagnose other associated conditions, similar to a premortem chest CT, despite changes to the lungs inherent with death.

## Patients

This study was approved by our National Research Ethical Committee (CONEP CAAE 30364720.0.0000.0068).

From March 2020 to early September 2021, 117 patients died because of laboratory-confirmed COVID-19 and underwent an autopsy that was requested by our institution’s medical staff, after informed consent was obtained from the next of kin. Of the 117 patients in our convenience sample, only 5 had a chest CT performed up to 2 days before death (4 patients in 2 days and one patient in one day) and a postmortem chest CT performed as part of a minimally invasive autopsy study. All patients were women, with a mean age of 36±20 years. The mean interval between death and the postmortem chest CT was 14 h 08 min±5 h 28 min. After the postmortem CT was performed, tissues from multiple organs were collected via ultrasound-guided biopsies.

A descriptive analysis is presented. Table 1 shows patient’s main data. Afterwards we describe each case with main clinical data and imaging findings.

**Figure 1:**
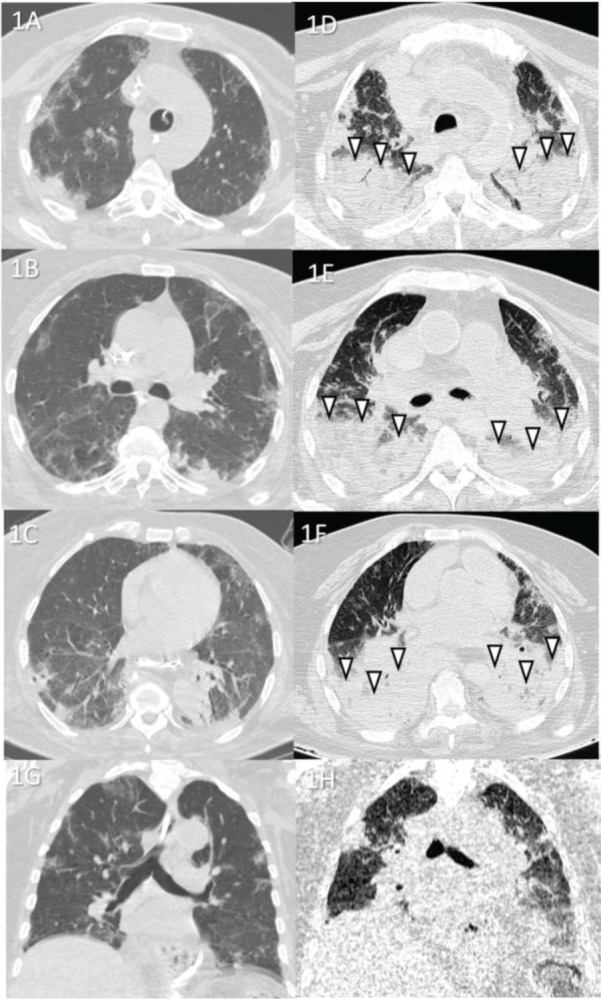
Case 1: Premortem axial chest computed tomography (CT) of the upper (A), mid (B), and inferior (C) thirds of the lungs obtained 2 days before death showing peripheral ground-glass opacities, slight consolidations, and interlobular septal thickening. Postmortem axial chest CT of the upper (E), mid (F), and inferior (G) thirds of the lungs obtained 4 h 47 min after death showing opacities larger than those on premortem CT, thus demonstrating the progression of the disease until death. Postmortem posterior “horizontal level forming” consolidations because of hypostasis (white arrowheads) are also observed, which limited analysis of the posterior lungs in this case (limitation of the method). Images D and H show pre- and postmortem coronal reformats, respectively.

**Case 1 (Figure 1)**: A 67-year-old female patient was hospitalized for approximately 1 month in the intensive care unit (ICU) before death. The cause of death was acute respiratory distress syndrome (ARDS) caused by COVID-19. Secondary pneumonia was also observed upon lung tissue analysis.

**Figure 2:**
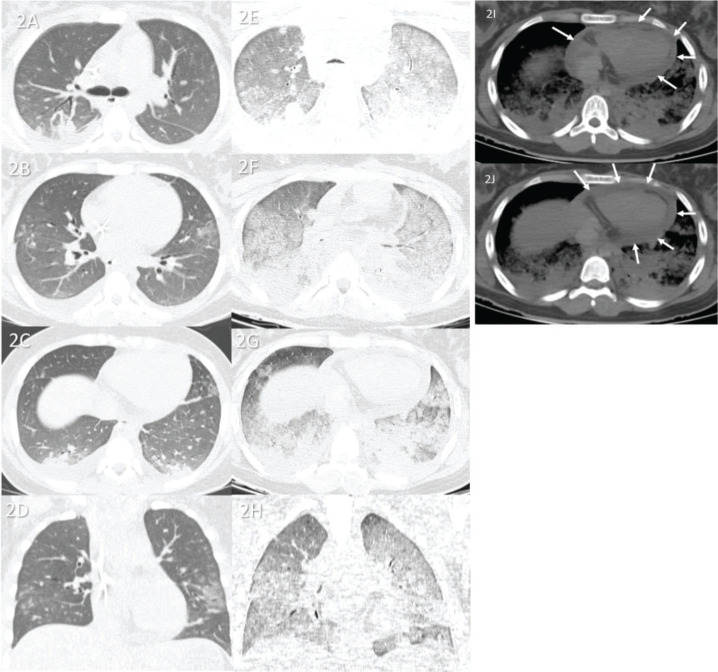
Case 2: Premortem axial chest computed tomography (CT) of the upper (A), mid (B), and inferior (C) thirds of the lungs obtained 2 days before death showing peripheral ground-glass opacities, small posterior bilateral consolidations, and right pleural effusion. Postmortem axial chest CT of the upper (E), mid (F), and inferior (G) thirds of the lungs obtained 14 h 03 min after death showing a diffuse “crazy paving” pattern, possibly because of acute respiratory distress syndrome and subsequent death. Furthermore, the posterior bilateral consolidations are larger on postmortem CT than on premortem CT, indicating progression of the disease. Images D and H show pre- and postmortem coronal reformats, respectively. Postmortem axial chest CT (I and J) of the mediastinal window showing pericardial effusion (white arrows) related to myopericarditis (confirmed with the collected tissue sample as the probable cause of death).

**Case 2 (Figure 2)**: An 11-year-old female patient with rapid progression of COVID-19 was admitted to the hospital 7 days after the onset of respiratory symptoms. She was directly admitted to the ICU and died 1 day later. The causes of death were myocarditis and ARDS caused by COVID-19. Lung tissue analysis revealed no secondary pneumonia.

**Figure 3:**
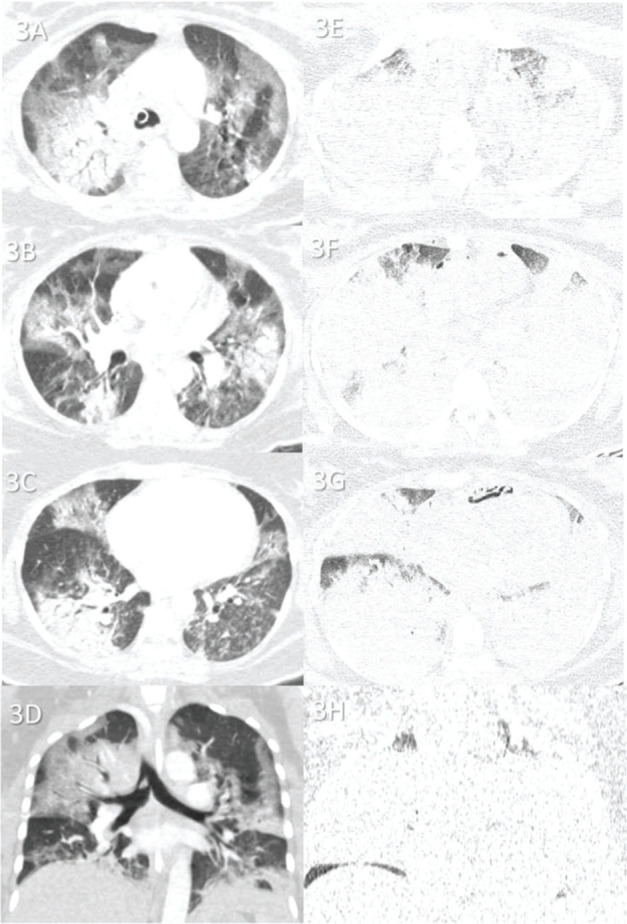
Case 3: Premortem axial chest computed tomography (CT) of the upper (A), mid (B), and inferior (C) thirds of the lungs obtained 2 days before death showing patchy peripheral and central ground-glass opacities and bilateral but mainly peripheral consolidations. Postmortem axial chest CT of the upper (E), mid, (F) and inferior (G) thirds of the lungs obtained 17 h 19 min after death showing rapid progression of the disease, with extensive consolidations in both lungs and a few areas of preserved pulmonary parenchyma. Images D and H show pre- and postmortem coronal reformats, respectively.

**Case 3 (Figure 3)**: A 35-year-old female patient was admitted to the hospital 5 days after the onset of respiratory symptoms. She was transferred to the ICU 4 days after admission and died 7 days later. The cause of death was ARDS caused by COVID-19. Secondary pneumonia was observed upon lung tissue analysis.

**Figure 4:**
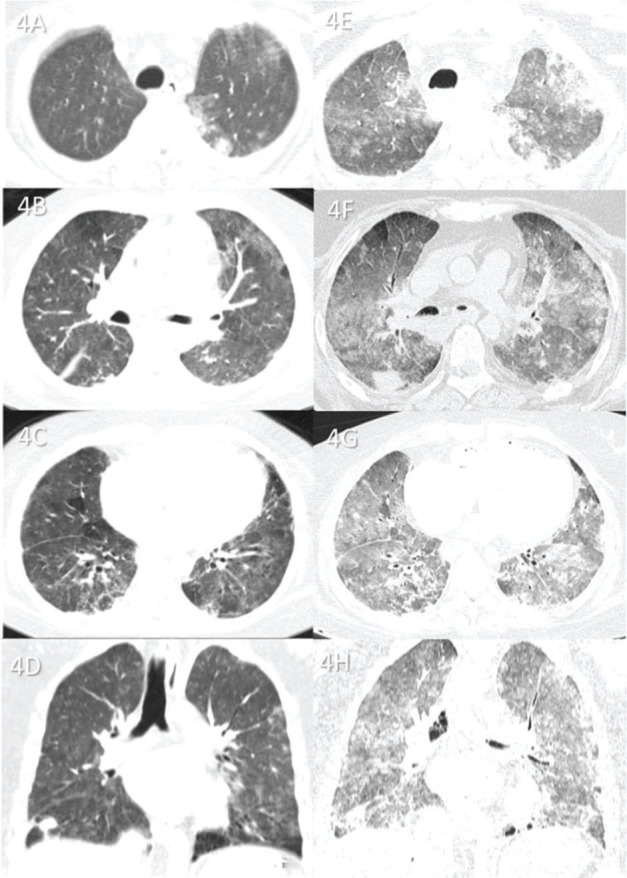
Case 4: Premortem axial chest computed tomography (CT) of the upper (A), mid (B), and inferior (C) thirds of the lungs obtained 1 day before death showing peripheral ground-glass opacities and diffuse pulmonary mosaic attenuation because of ventilation and/or perfusion disturbances (small hypoattenuating areas in the lungs). Postmortem axial chest CT of the upper (E), mid (F), and inferior (G) thirds of the lungs obtained 18 h 20 min after death showing ground-glass opacities larger than those on premortem CT and associated with small consolidations, indicating progression of the disease before death. Because the postmortem CT is of expired lungs, the pulmonary mosaic attenuation is enhanced. This confirms that the small hypoattenuating lung areas on premortem CT are air-trapping areas on postmortem CT. Images D and H show pre- and postmortem coronal reformats, respectively.

**Case 4 (Figure 4)**: A 38-year-old female patient was admitted to the ICU 9 days after the onset of respiratory symptoms and died 9 days later. The cause of death was ARDS caused by COVID-19. Lung tissue analysis revealed no secondary pneumonia.

**Figure 5:**
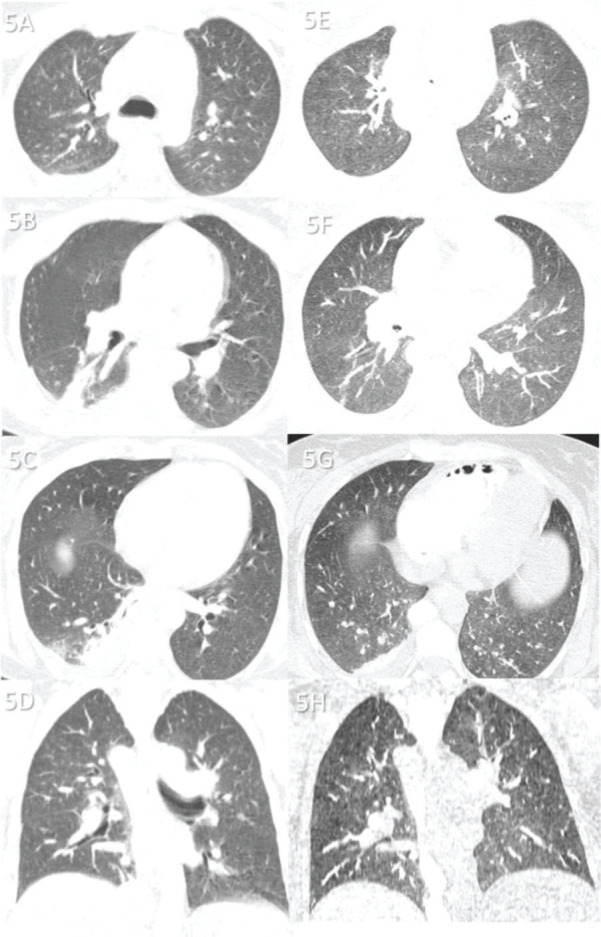
Case 5: Premortem axial chest computed tomography (CT) of the upper (A), mid (B), and inferior (C) thirds of the lungs obtained 2 days before death showing a few abnormalities, including a small peripheral and posterior ground-glass opacity in the lower right lobe (C), a small atelectasis in the posterior and medial aspects of the same lobe, and a right pleural effusion. Postmortem axial chest CT of the upper (E), mid (F), and inferior (G) thirds of the lungs obtained 16 h 13 min after death showing findings similar to premortem CT findings, except for a diffuse and subtle increase in attenuation of the lungs and thinning of the atelectasis in the right inferior lobe—changes probably because of the expired lungs during postmortem CT. The ground-glass opacity in the small right inferior lobe is not observed on postmortem CT, and the right pleural effusion is stable. Images D and H show pre- and postmortem coronal reformats, respectively. This patient died from liver transplant complications. Pre- and postmortem chest CT showing a normal lung parenchyma, indicating that she died with COVID-19, not from COVID-19.

**Case 5 (Figure 5)**: A 31-year-old female patient with a transplanted liver was admitted to the hospital because of hepatic complications. Nine days after admission, she developed respiratory symptoms and was transferred to the ICU, where she died 1 day later. The cause of death was acute liver failure. Lung tissue analysis revealed neither COVID-19-related pneumonia nor secondary pneumonia.

Table 2 presents the main positive and negative aspects of our analysis.

In all five cases, the logical radiological reasoning and interpretation of the main imaging findings showed disease progression until death. Despite the known limitations of postmortem CT, we were able to show that the information obtained can be useful in the appropriate scenario.

This study aimed to elaborate on the use of minimally invasive autopsy techniques, particularly in COVID-19 cases. The use of postmortem CT to help establish the correct cause of death is not new (12-15). During the COVID-19 pandemic, chest CT has played an important role in diagnosing and staging the disease in patients (16-18). Thus, it is logical to use postmortem CT to study COVID-related deaths, as we have attempted to do since the beginning of the COVID-19 pandemic. The supposed limitations of postmortem chest CT are already known: expired lungs (different from fully inspired lungs of the living); the dead can aspirate during their final moments; hypostasis may be present in the lungs (as in our case 1), depending on the time after death (19); and, of course, lung CT findings can change very quickly, within a few hours or days.

In addition, it is very difficult to identify a COVID-19 patient for whom a premortem chest CT was performed a few days before death, mainly because many of these patients are in severe clinical condition, with most in ICUs, which limits CT realization. Therefore, despite the relatively small number of cases, our results support the use of postmortem CT in this scenario.

Premortem chest CT findings were important for interpretation of postmortem CT findings. Our diagnostic performance improved when the findings were analyzed together. If we analyzed only postmortem CT findings of our cases, the evolution of the case would not be fully understood. Analysis of cases with almost fully consolidated lungs, such as our case 3, greatly benefits when a recent premortem CT is available for comparison. Furthermore, CT findings can be used to guide small tissue sample biopsies for important histopathologic analysis.

The major limitation of this study is the small number of cases. However, we hope this study inspires others to perform similar studies that add knowledge about minimally invasive autopsies being developed worldwide.

Postmortem chest CT can be useful in minimally invasive autopsies of fatal COVID-19 cases, especially if there is a recent premortem chest CT to compare with the postmortem CT and help interpret the findings. This interpretation can lead to logical diagnostic reasoning of the progression of COVID-19, and even reveal additional findings not related to SARS-CoV-2 infection, help understand the cause of death, and help guide small tissue sample biopsies, if necessary.

## Figures and Tables

**Table 1:** Main data of each patient.

Patient	Age (years)	Sex	Body mass index (kg/m^2^)	Time since symptoms onset until death (days)	Hospitalization time (days)	Days between pre- and postmortem CT	Time between death and postmortem CT	Cause of death^a^	Secondary pneumonia
1	67	F	32.6	32	29	2	4 h 47 min	ARDS/COVID-19	Yes
2	11	F	22.6	8	1	2	14 h 03 min	Myopericarditis/COVID-19	No
3	35	F	15.6	16	11	2	17 h 19 min	ARDS/COVID-19	Yes
4	38	F	20.4	18	9	1	18 h 20 min	ARDS/COVID-19	No
5	31	F	25.4	1	10	2	16 h 13 min	Acute liver failure	No

a Cause of death was determined via an histopathologic analysis of tissues collected through ultrasound-guided biopsy of multiple organs, performed after postmortem CT. ARDS, acute respiratory distress syndrome.

**Table 2:** Main positive and negative aspects of our analysis.

Positive aspects	Negative aspects
Confirms typical findings of COVID-19	Hypostasis (when present) may limit posterior lung analysis
Excludes typical findings of COVID-19	Expired lungs may obscure some findings
Determines progression of the disease	
Detects additional chest findings	
